# Severe Lipoatrophy in a Growth Hormone Deficient Toddler Girl Treated with a Non-Pegylated Long-Acting Growth Hormone

**DOI:** 10.3390/children12010058

**Published:** 2025-01-04

**Authors:** Atilla Büyükgebiz, And Demir

**Affiliations:** 1 Division of Pediatric Endocrinology, Department of Pediatrics, Demiroğlu Bilim University, İstanbul 34394, Türkiye; 2 Pediatric Research Center, New Children’s Hospital, University of Helsinki, Helsinki University Hospital, 00290 Helsinki, Finland; 3 Department of Pediatrics, Faculty of Medicine, Dokuz Eylül University, İzmir 35340, Türkiye

**Keywords:** long-acting growth hormone, non-pegylated, somatrogon, lipoatrophy, adverse event

## Abstract

Background: Recombinant growth hormone (rhGH) has been used since 1985 to treat growth hormone (GH)-induced short stature, typically associated with transient adverse events. However, lipoatrophy, characterized by irreversible damage to subcutaneous fat, was first reported in 1999 and linked to antibody formation. In 2021, localized lipoatrophy was observed in 14.5% of patients receiving daily rhGH, with repeated injections at the same sites being a common contributing factor. Long-acting rhGH (LAGH) preparation offers the advantage of weekly injections, enhancing patient comfort and adherence to treatment. Methods: This case report discusses a 5.5-year-old girl born at 40 weeks of gestation with a birth weight of 2300 g, diagnosed with idiopathic short stature and borderline GH secretion, along with a history of mild intrauterine growth retardation. Results: After initiating treatment with somatrogon, a non-pegylated fusion protein formulation of LAGH at the standard dose of 0.66 mg/kg body weight weekly, administered by her family, she developed localized lipoatrophy at the injection site within eleven weeks. The injections were performed consistently in the same area of the right upper arm, where lipoatrophy emerged. Following the onset of this adverse effect, her treatment was adjusted to daily rhGH, with strict instructions to rotate injection sites. Despite these clear instructions, follow-up revealed that the parents continued to administer injections with the non-pegylated LAGH fusion protein formulation, this time in the left upper arm, leading to a recurrence of lipoatrophy within eight weeks. Conclusions: The recurrence underscores the importance of proper injection techniques, particularly site rotation, in preventing localized adverse effects. Given the limitations of this case, where the recommended adjustments were not followed by the parents, it is crucial to emphasize that the administration of the preparation should be discontinued immediately upon the appearance of side effects such as lipoatrophy. Individual reactions to drugs are always possible, and this highlights the need for clinician vigilance in monitoring and addressing adverse effects promptly during treatments with LAGH.

## 1. Introduction

Recombinant human growth hormone (rhGH) therapy has been available for daily use since 1985. The expanded availability of rhGH has enabled a significant number of patients to receive treatment for GH-deficiency-induced short stature, as well as for non-GH-related short stature. Recently, Mameli et al. highlighted the importance of factors influencing the effectiveness of this therapy [1]. Several systemic and local adverse events have been reported, including the rare occurrence of lipoatrophy, which is generally transient [2,3,4].

Lipoatrophy is characterized by a significant depletion of subcutaneous fat, resulting in a visibly indented area on the skin. This condition stems from the destruction of underlying fat tissue [4,5]. Research suggests an immunological origin, as biopsies often reveal a high concentration of mast cells and eosinophils, indicating a localized inflammatory reaction that may drive tissue damage [5]. GH plays a role in promoting fat breakdown through direct stimulation of adipocytes and by reducing lipoprotein lipase activity, which decreases the transport of fatty acids into these cells [6]. Additionally, GH may contribute to reducing the number and size of fat cells, limiting the development of fat deposits, and decreasing overall body fat [7].

In 1999, we reported the first case of localized lipoatrophy due to rhGH therapy in a child with 6.7 kilobase gene deletion isolated growth hormone deficiency, and this lipoatrophy was irreversible, similar to that observed in insulin-treated type 1 diabetes patients [2]. In 2021, Chhiba and Segal published local lipoatrophy in 9 patients out of 62 between the ages of 5 and 12 who were receiving daily rhGH therapy in a single center, accounting for 14.5% of patients [3]. The development of localized lipoatrophy did not appear to be age, indication, or dose-related but rather related to repeated administration of rhGH into a limited number of sites. They hypothesized that one of the excipients involved in the production of rhGH could be causing lipoatrophy. Excipients, essential for enhancing stability and ensuring effective biomolecule delivery, must be evaluated for their biologic safety to enable patient-specific therapy customization, a critical consideration highlighted by earlier research [8]. Examples of adverse effects related to excipients include injection site reactions and anaphylaxis. The authors used the same brand of rhGH in all the patients. They used average doses of 25–50 µg/kg/day for the management of isolated GH deficiency, and the patients developed lipoatrophy at more than one injection site when more than one was used.

Long-acting GH (LAGH) therapies, including pegylated and non-pegylated forms, aim to improve compliance and clinical outcomes in children with growth hormone deficiency by reducing injection frequency [9]. Pegylation, attaching polyethylene glycol (PEG) to rhGH, extends the hormone’s half-life, allowing for weekly dosing [10]. Efforts to develop pegylated formulations have faced challenges in achieving adequate efficacy and safety profiles. For instance, ARX201 by Ambrx and PHA-794428 by Pfizer were discontinued due to significant concerns—PEG accumulation in the ependymal cells of the choroid plexus in primates for ARX201 and high rates of injection-site lipoatrophy for PHA-794428. Indeed, the 2009 study by Touraine et al. was the first to report injection-site lipoatrophy as a side effect observed in patients treated with long-acting pegylated growth hormone (PEG-LAGH), highlighting the importance of implementing site rotation strategies [6]. Lipoatrophy due to long-acting rhGH was observed in the phase 3 study. This study was designed to assess the safety and efficacy of a LAGH formulation created by covalently binding PEG to rhGH. The weekly subcutaneous formulation allowed for a progressive release of GH over up to 4 weeks [5]. According to the protocol, PEG-LAGH was to be injected into the same thigh to minimize variability. However, after five cases of lipoatrophy were identified, the trial involving 105 subjects was temporarily suspended and later resumed with an injection site rotation plan. Despite this adjustment, the emergence of additional cases, bringing the total to 13, including one in a pediatric participant, ultimately led to the study’s termination [6]. Søndergaard et al. also reported minimal side effects [11]. The most plausible explanation proposed was the direct lipolytic effect of GH on adipose tissue, supported by studies detailing GH’s impact on adipocytes and adipogenesis. Specifically, GH has been shown to stimulate lipolysis in mature adipocytes and primary preadipocytes while promoting adipogenesis in preadipocyte cell lines. This lipolytic activity arises from GH’s direct action on adipocytes and its suppression of lipoprotein lipase activity, which reduces the flow of free fatty acids into adipocytes. Consequently, GH appears capable of decreasing the volume of mature adipocytes, thereby limiting the expansion of adipose tissue and contributing to reductions in body fat [12]. In 2019, a team of researchers in China presented prospective data from a single-center study, documenting the outcomes of a two-year treatment with Jintrolong in children with growth hormone deficiency (GHD); their findings reinforced prior evidence, highlighting both the efficacy and safety of the treatment [13]. Recent reviews also reinforced that PEG-LAGH, such as Jintrolong, is as effective as daily injections in promoting growth. Zhu et al. reported similar or better height velocity scores and improved adherence due to fewer injections [14].

On the other hand, non-pegylated LAGH formulations employ various methods, such as binding to albumin or depot technology, among others [9]. Somapacitan is a once-weekly growth hormone formulation in which rhGH is conjugated to a fatty acid, enabling binding to serum albumin and thereby slowing its elimination. Sävendahl et al. reported injection site pain in only 3 out of 52 patients, with no lipodystrophy observed and no injection site pain reported in the switched group [15]. In a recent study, however, Rakusa et al. described a case involving a 38-year-old woman with congenital panhypopituitarism who experienced reduced subcutaneous tissue at all four injection sites after the fourth dose of somapacitan, leading to the discontinuation of treatment at the patient’s request, with complete resolution of lipoatrophy within three months [16].

To date, three non-pegylated LAGH analogs, developed using an alternative technology that involves fusing specific proteins to the rhGH molecule to extend its half-life and reduce immunogenicity, have advanced to phase 3 clinical trials in children with GHD. Among these, somatrogon (MOD-4023), a non-pegylated LAGH fusion protein developed by OPKO Health and Pfizer, integrates three copies of the C-terminal peptide from the β-subunit of human chorionic gonadotropin into the rhGH sequence [17]. Somatrogon, a weekly injectable, was found to be non-inferior to daily GH [18]. Zhu et al. noted similar efficacy to daily treatments, with variability in safety profiles [14]. A recent study by Zadik et al. [19] reported mild to moderate treatment-emergent adverse events in 81.3% of participants treated with somatrogon. Two participants discontinued the study due to adverse events—one due to scoliosis and the other due to osteochondrosis. No injection site reactions were reported. When a pen device was used for administration, three participants reported injection site reactions (bruising in two participants and erythema in one participant), which were mild to moderate in intensity. Notably, no cases of lipoatrophy were reported among the adverse events throughout the five-year study period.

In this report, we present a case involving a child who developed lipoatrophy following treatment with a non-pegylated LAGH fusion protein formulation that includes meta-cresol as a preservative. This case marks the first documented instance of lipoatrophy associated with a non-pegylated LAGH fusion protein formulation in the literature.

## 2. Case Report

### 2.1. Patient Information

The patient, a 5.5-year-old girl born at 40 weeks of gestation with a birth weight of 2300 g, was diagnosed with idiopathic short stature and borderline growth hormone (GH) secretion and has been followed up at our clinic.

### 2.2. Clinical Findings

At her initial evaluation at 5.5 years of age, the patient measured 99 cm in height and weighed 15.5 kg, placing her below the 3rd percentile (−2.3 SD) for height and at the 10th percentile for weight based on Turkish growth standards [20]. Thyroid function tests were within normal limits, and a clonidine-stimulated GH test revealed a peak GH level of 8.6 ng/mL. There was no evidence of GH resistance as her IGF-1 level was at the low-normal range for her age (75 ng/mL, reference range: 71–394 ng/mL), underscoring the need for careful monitoring to ensure adequate GH replacement therapy. IGF-1 levels serve as an essential biomarker for assessing the effectiveness of GH therapy and adjusting the dose to optimize growth while minimizing the risk of adverse effects. Additional laboratory assessments, including thyroid and celiac screenings, were unremarkable. No relevant medical, family, or psychosocial factors were identified as contributing to her current growth status. Before the initiation of treatment with the non-pegylated LAGH fusion protein formulation, the patient’s growth velocity was suboptimal.

### 2.3. Diagnostic Assessment

A clonidine-stimulated GH peak below 10 ng/mL, combined with a height below the 3rd percentile (−2.3 SD and a growth rate of 3.5 cm/year) —indicating failure to achieve catch-up growth by age 4—supported a diagnosis of idiopathic short stature with reduced GH secretion in this patient with a history of mild intrauterine growth retardation. These findings justified the initiation of GH therapy. The primary diagnostic challenge, however, was identifying the cause of the rapid onset of lipoatrophy following somatrogon administration, underscoring the need for early and vigilant monitoring and timely adjustments to the therapeutic regimen.

### 2.4. Therapeutic Intervention

**Figure 1:**
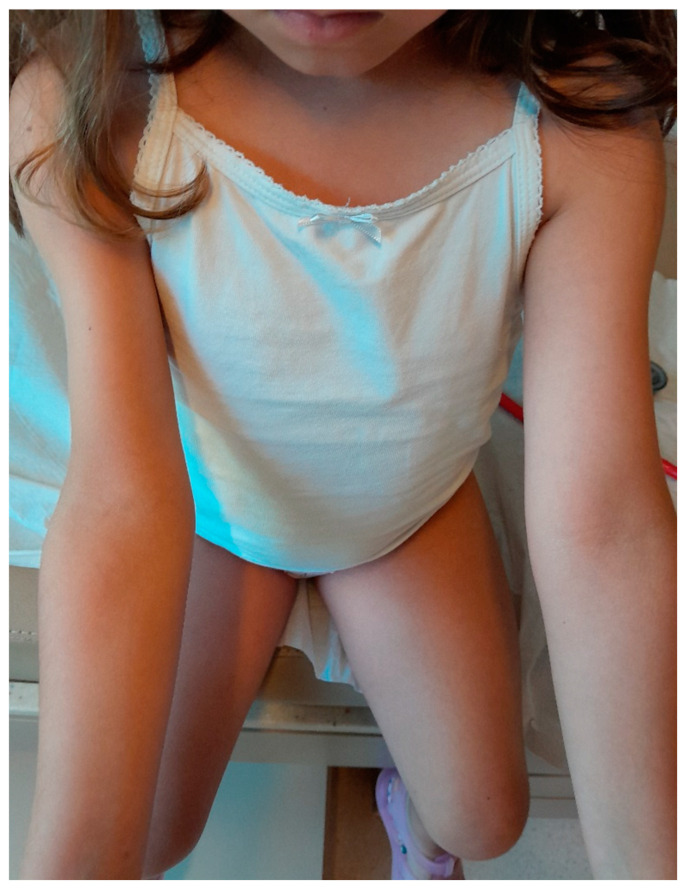
Lipoatrophy in the right upper arm after 11 weeks of weekly injections with the non-pegylated LAGH fusion protein formulation. The patient, a 5.5-year-old girl diagnosed with idiopathic short stature and growth retardation, developed lipoatrophy at the injection site due to repeated injections in the same localized area. The right upper arm mid-circumference measured 14 cm, compared to 16 cm in the left upper arm.

The patient initially received a weekly dose of 0.66 mg/kg body weight of the LAGH preparation Somatrogon (NGENLA, Pfizer, New York, NY, USA). Despite guidance on rotating injection sites, the parents consistently administered the injections in a limited area on the right upper arm. After 11 weeks of treatment, the patient developed lipoatrophy at the injection site, with the mid-circumference of the right upper arm measuring 14 cm compared to 16 cm on the left (Figure 1). Although the family reported adherence to the prescribed injection schedule, the lipoatrophy was attributed to repeated use of the same injection site on the right upper arm during this initial treatment period.

### 2.5. Follow-Up and Outcomes

**Figure 2:**
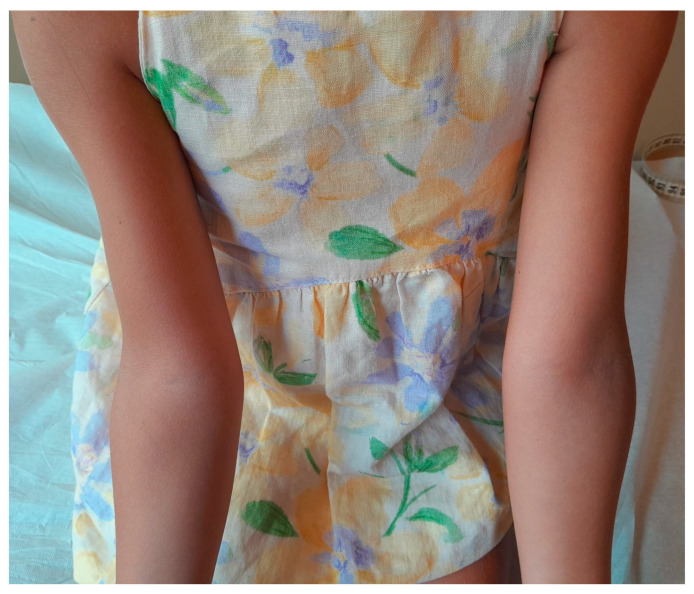
Progression of lipoatrophy in the left upper arm after an additional 8 weeks of using the non-pegylated LAGH fusion protein formulation during follow-up. Despite instructions to switch to daily hGH injections, the parents continued administering the non-pegylated LAGH fusion protein formulation in the left upper arm, resulting in further lipoatrophy. At the 8-week follow-up, the left upper arm mid-circumference had decreased to 14 cm, while the right upper arm showed slight recovery, with a mid-circumference of 14.5 cm.

Despite clear instructions to switch to daily injections of regular recombinant hGH with proper rotation of injection sites, a follow-up visit 8 weeks later revealed that the parents had continued administering somatrogon, now in the left upper arm. At the follow-up assessment at age 5.7 years, lipoatrophy recurred, with the mid-circumference of the left upper arm decreasing to 14 cm, while the right upper arm showed partial recovery, increasing to 14.5 cm (Figure 2). Despite this adverse reaction, the patient demonstrated a height increase of 3.5 cm over the treatment period, reflecting a positive response to the non-pegylated LAGH fusion protein formulation and confirming the initial diagnosis. Further evaluations indicated that her thyroid function and IGF-1 levels remained within normal ranges. The development of lipoatrophy in the left upper arm was attributed to repeated injections at the same site during the 8-week follow-up period.

### 2.6. Patient Perspective

The patient and her family experienced significant disappointment and distress following the development of lipoatrophy. Despite this challenging situation, they demonstrated a strong commitment to adhering to the treatment plan. However, a misunderstanding of the instructions led to continued administration of the medication in the other arm rather than transitioning to daily rhGH injections with proper rotation of injection sites as advised. Their willingness to adapt to the revised regimen, despite this initial misinterpretation, underscores their dedication to effectively managing the patient’s condition.

### 2.7. Informed Consent

Informed consent was obtained from both the patient and her guardians for the publication of this case report, including permission to share detailed case information, images, and clinical outcomes.

## 3. Discussion

This is the first documented case of lipoatrophy associated with a non-pegylated LAGH fusion protein formulation, observed in a child treated with a weekly rhGH containing meta-cresol as a preservative.

Notably, the lipoatrophy developed within 8 to 11 weeks, indicating a rapid onset. Although severe cutaneous reactions to insulin due to meta-cresol sensitivity have been reported, no prior cases of lipoatrophy associated with this preservative have been documented in the context of rhGH therapy [21,22].

Meta-cresol’s mild irritant properties and cytotoxic effects on adipocytes may contribute to localized fat cell apoptosis, particularly when injections are repeatedly administered at the same site. Additionally, the glycosylated C-terminal peptides (CTPs) incorporated into the non-pegylated LAGH fusion protein formulation to extend its half-life may provoke localized immune responses, leading to inflammation and fat cell degradation. The combination of these factors, alongside the extended retention time of the formulation at the injection site, likely contributed to the development of lipoatrophy in this case.

In this case, the non-pegylated LAGH fusion protein formulation incorporates glycosylated C-terminal peptides (CTPs) derived from human chorionic gonadotropin to extend its half-life. However, glycosylation modifies the protein’s structure, potentially triggering localized immune responses and inflammation at the injection site. This chronic inflammatory response may contribute to fat cell degradation and tissue remodeling. Given the long-acting feature of the non-pegylated LAGH fusion protein formulation, the extended presence of the injected material at the site may increase the likelihood of localized irritation and inflammation.

We hypothesize that the lipoatrophy observed, in this case, may result from the combined effects of glycosylated CTPs in the non-pegylated LAGH fusion protein formulation, which may cause mild irritation, and the cytotoxic impact of meta-cresol on adipocytes, particularly with prolonged exposure at the same injection site. In this case, we attribute the development of lipoatrophy primarily to insufficient rotation of the injection site.

LAGH preparations were developed to improve patient compliance and adherence to treatment regimens, as well as to enhance the quality of life for children undergoing GH therapy [1]. The non-pegylated LAGH fusion protein formulation, in this case, is a long-acting formulation that shares the same amino acid sequence as rhGH, with an added carboxy-terminal peptide derived from human chorionic gonadotropin. In a comparative study by Deal et al., the growth-promoting effects of rhGH and the non-pegylated LAGH fusion protein formulation were evaluated, showing similar efficacy between the two. However, the non-pegylated LAGH fusion protein formulation was associated with a higher incidence of adverse effects, such as pruritus and pain, with treatment in one patient discontinued due to injection site pain [18]. Conversely, a recent systematic review and meta-analysis by Zhu et al., which examined four LAGH preparations, concluded that these formulations generally had fewer adverse effects than daily GH injections, although some variations were noted across the different preparations [14].

Our case report has several limitations that should be acknowledged. As a single case study, the findings are based on a limited sample size, which restricts generalizability to a broader patient population. Additionally, the absence of antibody testing for hGH precluded exploration of potential immunological mechanisms contributing to lipoatrophy, leaving an important aspect unaddressed. The relatively short follow-up period may also limit insights into longer-term outcomes, such as whether the lipoatrophy persisted or resolved over time. Furthermore, the data was collected from a single clinical setting, which may not reflect variations in patient demographics or treatment practices that could affect the occurrence of lipoatrophy. Also, caregivers’ reliance on self-reported adherence to proper injection techniques introduces uncertainty, as direct observation was lacking to verify these practices, potentially impacting the accuracy of our conclusions. Finally, it should be noted that the female patient was small for gestational age without catch-up and did not have a typical growth hormone deficiency.

Based on our observations, several strategies may optimize the treatment and management of lipoatrophy in patients receiving LAGH treatment. Continuous monitoring is essential, as regular follow-up enables early detection and timely intervention, allowing for treatment adjustments before the condition advances. Educating patients and caregivers on correct injection techniques, with an emphasis on rotating injection sites, is crucial to mitigating the risk of lipoatrophy. Ensuring that caregivers are well-informed about proper injection practices can significantly reduce the likelihood of adverse skin reactions.

Finally, we emphasize that the administration of the preparation should be discontinued immediately in the event of side effects such as lipoatrophy. It is important to recognize that individual reactions to drugs vary, and it is neither appropriate nor scientifically sound to generalize or discourage the use of this medication broadly based on a single case. Instead, a balanced approach involving vigilant monitoring, education, and tailored treatment adjustments can ensure patient safety while preserving the therapeutic benefits of LAGH formulations.

## 4. Conclusions

Lipoatrophy has been shown to be linked to non-pegylated LAGH fusion protein formulation in this particular case report; however, it is essential to acknowledge that individual responses to such treatments vary, and broad generalizations or discouraging statements about their use should be avoided. Further research is warranted to explore potential links between lipodystrophy and structural modifications in growth hormone molecules, refine dosing strategies, and optimize management protocols.

A comprehensive approach remains essential, including continuous patient monitoring, education on proper injection techniques with an emphasis on site rotation, and careful dosage adjustments, particularly for prepubertal children and those with a slimmer build. In cases where adverse effects such as lipoatrophy arise, immediate discontinuation of the preparation is necessary to prevent further complications.

## Data Availability

The data presented in this study are available on request from the corresponding author. The data are not publicly available due to ethical reasons.
